# Epidemiology, clinical characteristics and risk factors of coronavirus disease 2019 (COVID-19) in Casablanca

**DOI:** 10.1099/acmi.0.000400

**Published:** 2023-04-21

**Authors:** Soulandi Djorwé, Amale Bousfiha, Néhémie Nzoyikorera, Victor Nkurunziza, Khadija Ait Mouss, Bellamine Kawthar, Abderrahim Malki

**Affiliations:** ^1^​ Laboratory of Physiopathology and Molecular Genetics, Faculty of Sciences Ben M'Sik, Hassan II University of Casablanca (Morocco), Avenue Cdt Driss El Harti, PB 7955 Sidi Othman Casablanca, Morocco; ^2^​ Bourgogne Laboratory of Medical and Scientific Analysis, 136, Residence Belhcen, Bd Bourgogne, Casablanca, Morocco; ^3^​ National Reference Laboratory, National Institute of Public Health, Bujumbura, Burundi; ^4^​ Higher Institute of Biosciences and Biotechnology, Mohammed VI University of Health Sciences (UM6SS), Casablanca, Morocco; ^5^​ Laboratory of Microbial Biotechnology and Infectiology Research, Mohammed VI Center for Research & Innovation, Rabat, Mohammed VI University of Health Sciences (UM6SS), Casablanca, Morocco; ^6^​ Laboratory of Hematology, University Hospital Centre Ibn Rochd, 1, Rue des Hôpitaux, 20100, Casablanca, Morocco; ^7^​ Department of Microbiology, Faculty of Medicine and Pharmacy, Hassan II University of Casablanca, 19 rue Tarik Bnou Zyad, 20360, Casablanca, Morocco

**Keywords:** COVID-19, clinical manifestations, risk factors, RT-PCR, SARS-CoV-2

## Abstract

This is an analytical cross-sectional study of coronavirus disease 2019 (COVID-19) based on data collected between 1 November 2020 and 31 March 2021 in Casablanca focusing on the disease’s epidemiological status and risk factors. A total of 4569 samples were collected and analysed by reverse-transcription polymerase chain reaction (RT-PCR); 967 patients were positive, representing a prevalence of 21.2 % for severe acute respiratory syndrome coronavirus 2 (SARS-CoV-2). The mean age was 47.5±18 years, and infection was more common in young adults (<60 years). However, all age groups were at risk of COVID-19, and in terms of disease severity, the elderly were at greater risk because of potential underlying health problems. Among the clinical signs reported in this study, loss of taste and/or smell, fever, cough and fatigue were highly significant predictors of a positive COVID-19 test result (*P<*0.001). An assessment of the reported symptoms revealed that 27 % of COVID-19-positive patients (*n*=261) experienced loss of taste and/or smell, whereas only 2 % (*n*=72) of COVID-19-negative patients did (*P<*0.001). This result was consistent between univariate (OR=18.125) and multivariate (adjusted OR=10.484) logistic regression analyses, indicating that loss of taste and/or smell is associated with a more than 10-fold higher multivariate adjusted probability of a positive COVID-19 test (adjusted OR=10.48; *P<*0.001). Binary logistic regression model analysis based on clinical signs revealed that loss of taste and/or smell had a performance index of 0.846 with a *P<*0.001, confirming the diagnostic utility of this symptom for the prediction of COVID-19-positive status. In conclusion, symptom evaluation and a RT-PCR [taking into account cycle threshold (*C*_t_) values of the PCR proxy] test remain the most useful screening tools for diagnosing COVID-19. However, loss of taste/smell, fatigue, fever and cough remain the strongest independent predictors of a positive COVID-19 result.

## Data Summary

All data used in this study were extracted from the LIS (a specialized platform for managing COVID-19 analyses created by the Moroccan Ministry of Health and designed by ENOVA Research and Technology in 2020) and are available as Excel files with the article. Supplementary data files can be found at: 10.6084/m9.figshare.19358135 [1].

## Introduction

On 31 December 2019, the World Health Organization (WHO) office in PR China was informed of an episode of viral pneumonia of unknown aetiology in Wuhan, PR China, heralding the onset of coronavirus disease 2019 (COVID-19) [2, 3]. Forty-four cases were reported to WHO on 31 December 2019. On 11 January 2020, extensive research identified and even isolated the causative agent as a new type of coronavirus. Shortly there after, PR China shared the genetic sequence of the new coronavirus with other countries, allowing the development of specific diagnostic kits [2, 4, 5]. Initially, the pathogen was tentatively named 2019 novel coronavirus (2019-nCoV), but it was later renamed severe acute respiratory syndrome 2 (SARS-CoV-2) by the International Committee on Taxonomy of Viruses (ICTV) [6]. COVID-19 was the name given to the disease caused by SARS-CoV-2. Since the first reported cases in December 2019, the international epidemiological situation as of 28 March 2021 was 126 372 442 confirmed COVID-19 cases, including 2 769 696 deaths in more than 200 countries and territories, including Morocco [7, 8]. The number of cases recorded in the African continent was not as alarming as those recorded in other continents.

In Morocco, the first case was confirmed on 2 March 2020 but numbers increased rapidly and reached 623 528 cases with 9785 deaths on 31 July 2021 [9, 10]. During the period of this study, two [3] variants were circulating locally in Morocco: the alpha variant known as lineage 20B/501Y.V1, VOC 202012/01 or B.1.1.7, and the beta variant known as 501Y.V2[11, 12]. Faced with this imminent threat, a response plan was established. This involved the declaration of a state of health emergency throughout the country, with the closure of borders, schools and universities, a ban on inter-city travel and a nationwide lockdown. The Ministry of Health, in collaboration with its technical partners (laboratories equipped with diagnostic tools and qualified professionals), drew up a response plan, the implementation of which involved both researchers and health system actors. The severity of SARS-CoV-2 infection ranges from mild or moderate to severe or critical, sometimes requiring hospitalization [13, 14]. SARS-CoV-2 belongs to the family of viruses capable of causing multiple symptoms. The Centers for Disease Control and Prevention (CDC) in the USA has listed the following symptoms: fever or chills, cough, shortness of breath or difficulty breathing, fatigue, muscle or body aches, headache, loss of taste and/or smell, throat irritation, nasal congestion, nausea or vomiting, and diarrhoea [15]. As the pandemic progressed, several other symptoms were reported in the literature, including those reported in the Cochrane Library’s documented reviews of 27 signs and symptoms in 4 different categories: systemic, respiratory, gastrointestinal and cardiovascular, all of which concerned patients who tested positive for COVID-19 [14].

According to WHO, presumed active SARS-CoV-2 infections should be screened by nucleic acid amplification tests (NAATs), including reverse-transcription polymerase chain reaction (RT-PCR). These tests can differentiate between pneumonia caused by SARS-CoV-2 and pneumonia caused by other types of viruses [14, 16]. In the same vein, WHO has emphasized that decisions regarding coronavirus disease must be backed up by evidence and scientific data [17]. It is therefore imperative that the epidemiology and clinical characteristics of confirmed cases are provided in sufficient detail to establish key guidelines on how to contain the threat. Prior knowledge of the epidemic characteristics and functional clinical signs of COVID-19 will assist in the establishment of an optimal action plan to control the epidemic.

In this context, this study aims to examine and analyse the epidemiological and clinical profile of COVID-19, especially with respect to the factors suggesting SARS-CoV-2 infection, in one of the COVID-19 screening centres in Casablanca, Morocco. Furthermore, the diagnostic performance of some viral genome amplification kits that use RT-PCR was tested and the prevalence of COVID-19 genes (*E*, *N* and *RdRp*) in COVID-19-positive Moroccan subjects was estimated while taking into account the importance of the interpretation of the cycle threshold (*C*_t_) during SARS-CoV-2 infection.

## Methods

### Study population and sampling

This is a retrospective analytical study that was conducted on subjects wishing to be screened for various reasons: either because they suspected they had a clinical sign of COVID-19 infection, or because they had been in recent contact with COVID-19-positive subjects, or because they wanted to travel, or for other miscellaneous reasons. This study was conducted over a period of 5 months from 1 November 2020 to 31 March 2021. The samples were obtained using nasopharyngeal swabs and were collected in tubes containing stable universal viral transport media (Nal von MindenGmbH, Germany) after recording clinical and demographic information. All samples received were processed in a room equipped with a level 2 biosafety facility, according to the required standards of the molecular biology unit. All samples were accompanied by information forms for the performance of a virological examination of COVID-19 by RT-PCR. The information form included the following information: patient ID, gender, age, reason for testing, presence of signs or symptoms. A patient was classified as asymptomatic if he or she was infected with SARS-CoV-2, but did not have symptoms of COVID-19, while a symptomatic patient had signs and symptoms of COVID-19. All information was entered into LIS (a specialized platform for the management of COVID-19 analyses set up by the Moroccan Ministry of Health and designed by ENOVA Research and Technology in 2020). The final database used was extracted from LIS in Excel format.

### RNA extraction

Two systems (automatons) were used for viral RNA extraction and purification; the magnetic particle processor Thermo Scientific KingFisher Duo Prime (USA) and Nextractor NX-48SGenolution (Republic of Korea) according to the manufacturer’s recommendations.

### Detection of SARS-CoV-2 RNA by RT-PCR

The presence of SARS-CoV-2 was confirmed using Argene amplification kits (bioMérieux SA., France) and GeneProof kits (GeneProof a.s., Czech Republic) by amplification of the regions of *RdRp*, *E* and *N* genes; the kits are designed to detect SARS-CoV-2 RNA qualitatively by transcription and PCR amplification of specific regions of the SARS-CoV-2 target genome. The limit of detection of the Argene amplification kit is 380 copies ml^−1^ of SARS-CoV-2 viral genome RNA, while that of the GeneProof kit is 519 copies ml^−1^. The criteria for interpreting the results of the kits used were as follows.

Argene kit:

- A sample was only considered positive if the *C*_t_ values of either or both genes were <40;
- Positive control (PC) should give a signal (*C*_t_) lower than or equal to 34;
- Internal control (IC) should give a signal (*C*_t_) lower than or equal to 36.

GeneProof kit:

- A sample was only considered positive if the *C*_t_ values of either or both genes were <40;
- Presence of internal control (IC).

For each kit, in order to ensure the integrity and verification of the test results, the internal control is run in parallel for each patient sample, in addition to the inclusion of positive and negative controls, which are required for the test to be valid. All samples with a *C*_t_ of 40 and above were considered negative. All reactions were run on QuantStudio 5 (Applied Biosystems, USA) with QuantStudio Design and Analysis Software version 1.5.1. The results were interpreted according to the manufacturer’s instructions. The viral loads were classified into three groups: 10≤*C*_t_<20 indicates high viral load; *C*_t_ value 20–30 indicates moderate viral load; and *C*_t_ value >30 indicates low viral load [18–22].

### Statistical analysis

In this study, continuous variables were presented as medians and means±standard deviation, and interquartile ranges were calculated for the normal and skewed data distributions. Comparisons between different categorical variables are presented as numbers and percentages and evaluated by Fisher’s exact test, the chi-square test, or the Mann–Whitney U test. Spearman’s and Pearson’s correlation tests were performed to calculate the correlation between the different types of variables. Univariate and multivariate analyses based on the logistic regression method were performed to determine the odd ratio (OR) and adjusted OR. *P*-values <0.05 were considered statistically significant and those <0.001 were considered highly significant. The results of statistical analyses and graphs were generated using IBM SPSS Statistics v25. The table showing correlation between clinical signs was generated using Rx64 software v4.0.4. The scatter plots and whisker boxes were generated using GraphPad Prism 8 for OS X (version 8.0.1; LLC).

## Results

### General characteristics of the COVID-19 cases detected

**Fig. 1.**
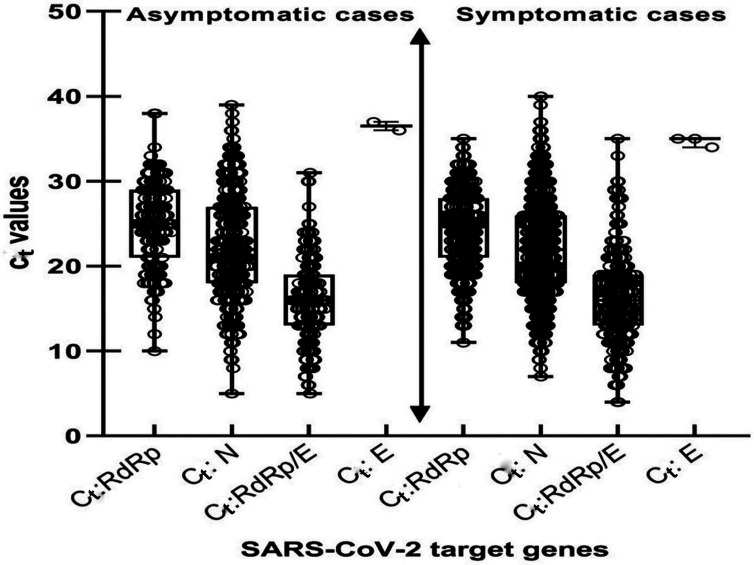
Distribution of asymptomatic and symptomatic cases of COVID-19-positive patients based on which SARS-CoV-2 gene was targeted by PCR. Boxplot showing the distribution of *C*_t_ values obtained for the *RdRp*, *N* and *E* genes targeted by the Argene kit and the *RdRp/E* and *N* genes targeted by the GeneProof kit. The *y*-axis shows the *C*_t_ values. The centre lines of the whisker boxes represent median *C*_t_ values; the limits of the box plots indicate the first and third quartiles. The number of data points is displayed in each dataset.

A total of 4569 samples were collected to search for SARS-CoV-2 by RT-PCR between 1 November 2020 and 31 March 2021, of which 2369 (51.8 %) were from women and 2200 (48.2 %) were from men. The samples were collected in the Casablanca-Settat region. Of the 4569 patients examined, 3352 (73.4 %) were asymptomatic cases, which was significantly (*P*<0.001) higher (73.4 %, *n*=3352) than symptomatic cases (26.6 %; *n*=1217). Among the analysed samples, 967 out of 4569 samples were positive, giving a prevalence rate of 21.2 % (95 % CI=20–22.4 %). The mean age±standard deviation of the COVID-19-positive patients was 47.5±18 years (range: 3–91 years), with a median age of 48 years. By contrast, the mean age±standard deviation of the COVID-19-negative patients was 42.3±17.5 years (range: 2–97 years), with a median age of 40 years. Among the 4569 samples analysed, most were obtained from the 31–40 (20.5 %) age group, whereas few were obtained from the ≤10 (0.7 %) and 91–100 (0.2 %) age groups (Table 1). In general, the distribution of age by sex was approximately the same (Mann–Whitney test, *P*<0.05). However, there was a significant difference in the proportion of COVID-19-positive patients between the sexes (Mann–Whitney test, *P<*0.001). Furthermore, among the 967 confirmed cases, 66.2 % (*n*=640) were symptomatic cases and 33.8 % (*n*=327) were asymptomatic cases, demonstrating a high percentage of symptomatic cases [95 % CI=32.4 % (25.91–38.46 %); *P<*0.001]. Among 4569 samples analysed, 34 were children, with 79.4 % (*n*=27) asymptomatic cases and 20.6 % (*n*=7) symptomatic cases identified. Among the five positive cases in children, all were asymptomatic. In terms of symptom categories, patients with the smallest *C*_t_ values (10≤*C*_t_<20) were recorded in symptomatic subjects, showing that these subjects had a statistically higher viral load than asymptomatic subjects (*P<*0.001) (Fig. 1).

**Table 1.** General characteristics of the subjects tested and their SARS-CoV-2 infection status

Associated factors	COVID-19-positive	COVID-19-negative	Total	***P***-value
TOTAL	967	3602	4569	
**Sex of the subjects**				*P<*0.001
Male	414 (42.8 %)	1786 (49.6 %)	2200 (48.2 %)	
Female	553 (57.2 %)	1816 (50.4 %)	2369 (51.8 %)	
**Age groups**				
≤10	5 (0.5 %)	29 (0.8 %)	34 (0.7 %)	*P=*0.33
11 to 20	43 (4.4 %)	239 (6.6 %)	282 (6.1 %)	*P<*0.05
21 to 30	134 (13.8 %)	736 (20. 4 %)	870 (19 %)	*P<*0.001
31 to 40	171 (17.6 %)	766 (21.2 %)	937 (20.5 %)	*P<*0.05
41 to 50	159 (16.4 %)	621 (17.2 %)	780 (17 %)	*P=*0.55
51 to 60	185 (19.1 %)	565 (15.6 %)	750 (16.4 %)	*P<*0.05
61 to 70	151 (15.6 %)	365 (10.1 %)	516 (11.3 %)	*P<*0.001
71 to 80	87 (9 %)	194 (5.4 %)	281 (6.1 %)	*P<*0.001
81 to 90	30 (3.1 %)	78 (2.1 %)	108 (2.3 %)	*P=*0.06
91 to 100	2 (0.2 %)	9 (0.2 %)	11 (0.2 %)	–
**Fever**				*P<*0.001
Yes	342 (35.4 %)	164 (4.6 %)	506 (11.1 %)	
No	625 (64.6 %)	3438 (95.4 %)	4063 (88.9 %)	
**Fatigue**				*P<*0.001
Yes	429 (44.4 %)	365 (10 %)	794 (17.4 %)	
No	538 (55.6 %)	3237 (90 %)	3775 (82.6 %)	
**Cough**				*P<*0.001
Yes	286 (29.6 %)	218 (6 %)	504 (11 %)	
No	681 (70.4 %)	3384 (94 %)	4065 (89 %)	
**Diarrhoea**				*P<*0.001
Yes	99 (10.2 %)	78 (2 %)	177 (3.9 %)	
No	868 (89.8 %)	3524 (98 %)	4392 (96.1 %)	
**Loss of taste and/or smell**				*P<*0.001
Yes	261 (27 %)	72 (2 %)	333 (7.3 %)	
No	706 (73 %)	3530 (98 %)	4236 (92.7 %)	
**Others symptoms**				*P<*0.001
Yes	134 (14 %)	166 (4.6 %)	300 (6.6 %)	
No	833 (86 %)	3436 (95.4 %)	4269 (93.4 %)	
**Reason for testing**				
**Contact cases**				*P<*0.001
Yes	341 (35.3 %)	983 (27.3 %)	1324 (29 %)	
No	626 (64.7 %)	2619 (72.7 %)	3245 (71 %)	
**Screening**				*P<*0.001
Yes	571 (59 %)	1105 (30.7 %)	1676 (36.7 %)	
No	396 (41 %)	2497 (69.3 %)	2893 (63.3 %)	
**Travel**				*P<*0.001
Yes	20 (2.1)	1368 (38 %)	1388 (30.4 %)	
No	947 (97.9 %)	2234 (62 %)	3181 (69.6 %)	
**Others**				*P<*0.001
Yes	35 (3.6 %)	146 (4 %)	181 (4 %)	
No	932 (96.4 %)	3456 (96 %)	4388 (96 %)	

### Distribution of SARS-CoV-2 according to the variables collected

Table 1 shows the distribution of SARS-CoV-2-positive cases in our study sample. The number of infections among women (*n*=553; 57.2 %) was higher than among men (*n*=414; 42.8 %). There was a significant difference in the positivity rate between men and women [95 % CI: 14.4 % (10–18.7 %); *P*<0.001]. The largest number of samples were from the age group 51–60 years (19.1 %). The age groups ≤10 years (0.5 %) and 91–100 years (0.2 %) were less prone to COVID-19.

**Fig. 2.**
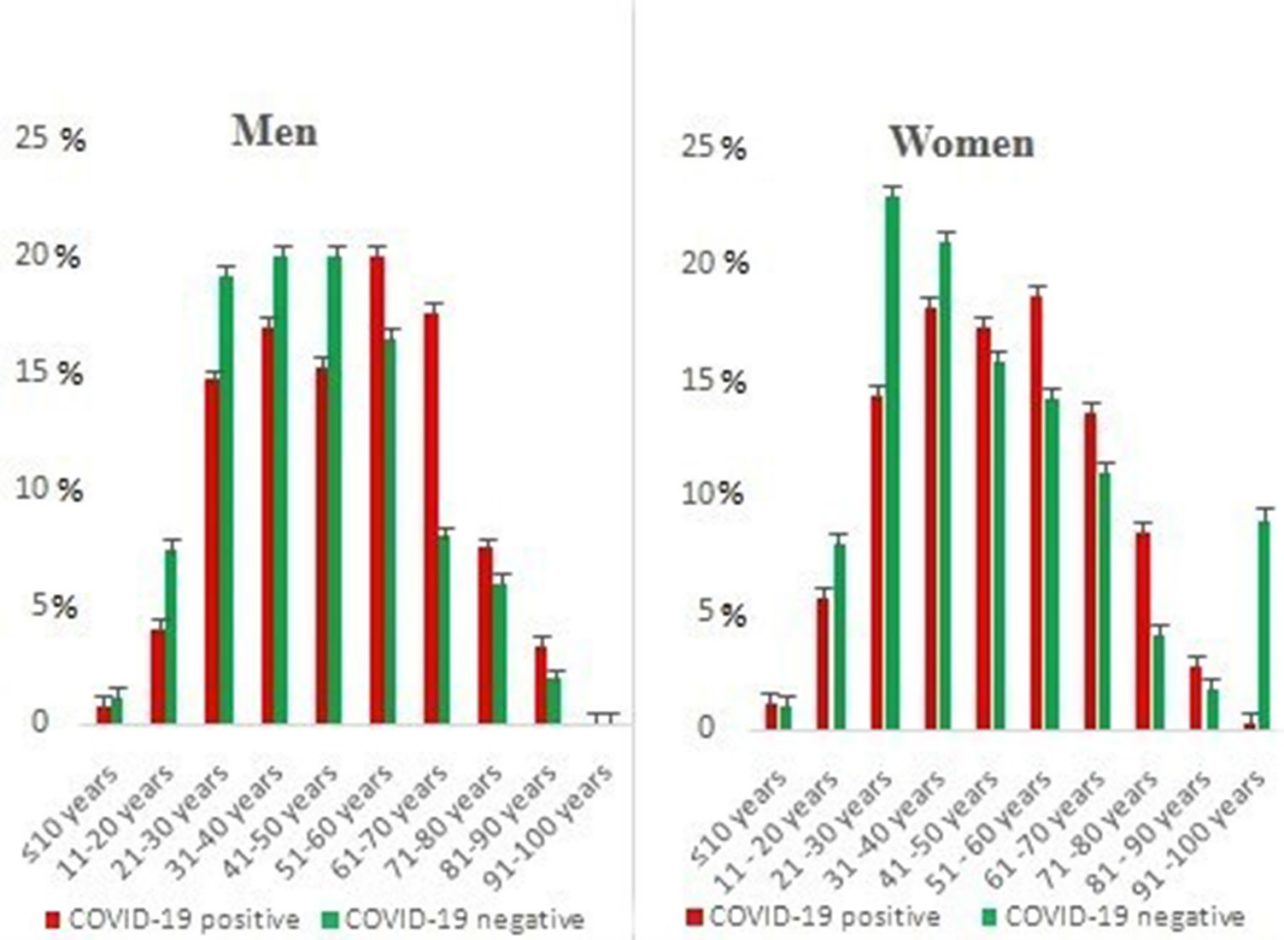
Distribution of SARS-CoV-2 infection cases according to age and sex.

Among all recorded SARS-CoV-2-positive cases, the highest number of infections was 19.1 % (*n*=185) in the age group 51–60 years, followed by 17.6 % (*n*=171) in the age group 31–40 years. The smallest number of cases was in the age group ≤10 years, with 0.5 % (*n*=5) of cases and those of 91–100 years with 0.2 % (*n*=2). The lowest rate (0.2 %; *n*=1) for men and women was observed in the age group 91–100 years, followed by those in the age group ≤10 years (1.1 %; *n*=6) (Fig. 2). Linear regression showed a highly significant association between age, sex, and SARS-CoV-2 infection (*P<*0.001).We divided symptomatic patients according to the following functional signs: fatigue was the most reported symptom, followed by fever, cough, loss of taste and/or smell (Table 1).

### Disease symptom clustering

**Fig. 3.**
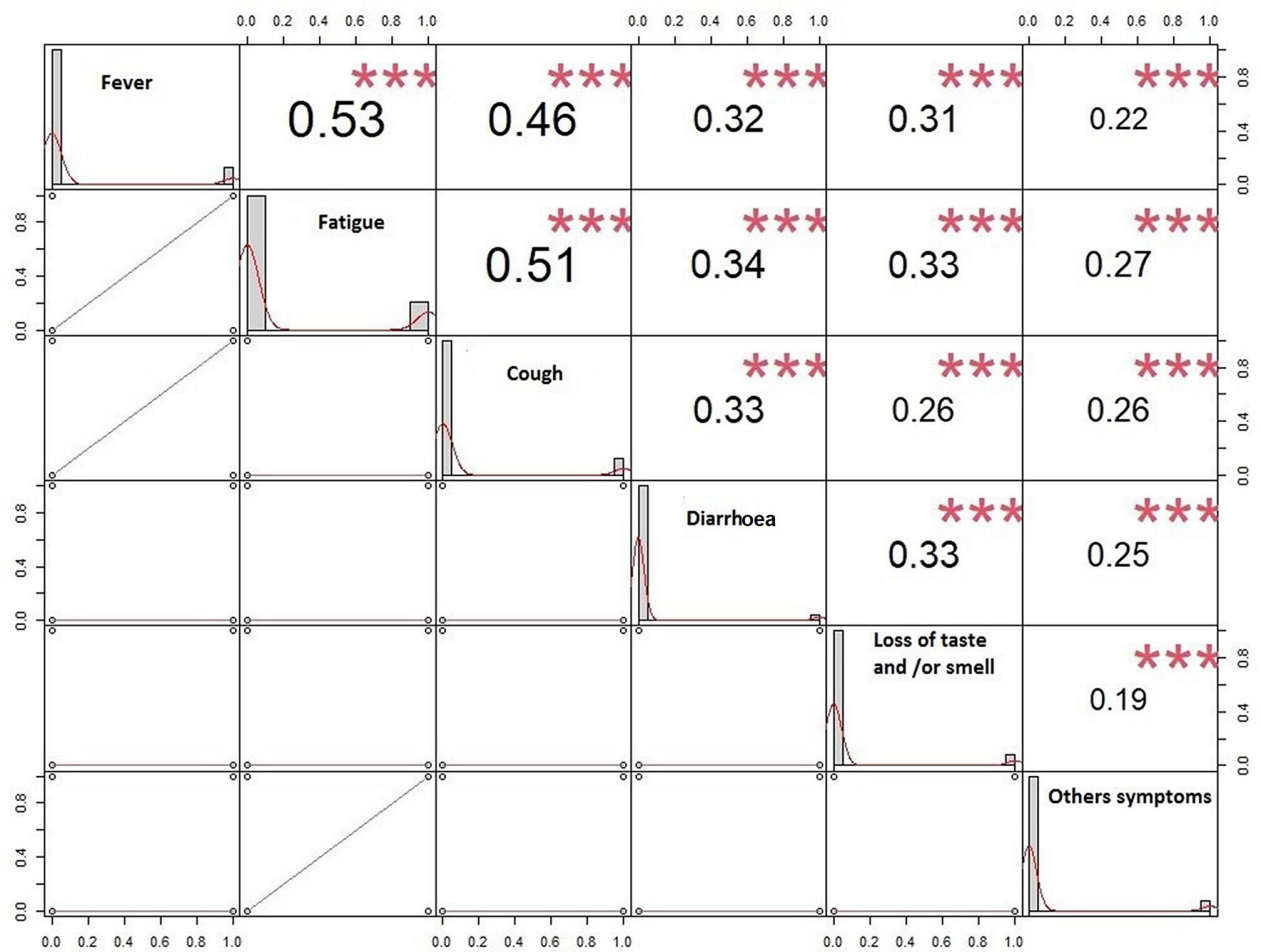
Pairwise correlations between the clinical symptoms of positive and negative cases. The diagonal shows the distribution of the variables with the trendline. The bottom of the diagonal shows the regression lines between the different variables. The top of the diagonal shows correlation coefficients and significance levels (red stars). *P*-values (***, <0.001; **, <0.01; *, <0.05).

In order to identify symptom clusters (combinations of symptoms) that may indicate a probable COVID-19 infection, we performed pairwise Pearson correlations of patient symptoms (Fig. 3). The Pearson’s r (correlation coefficient) values between −1 and 1 were used to identify relationships between variables (symptoms) and trend curves and regression lines were generated. Correlation analysis revealed moderate correlations between symptoms, which were highly significant (*P<*0.001) between fever and fatigue (r=0.53), fatigue and cough (r=0.51), and fever and cough (r=0.46) (Fig. 3).

**Fig. 4.**
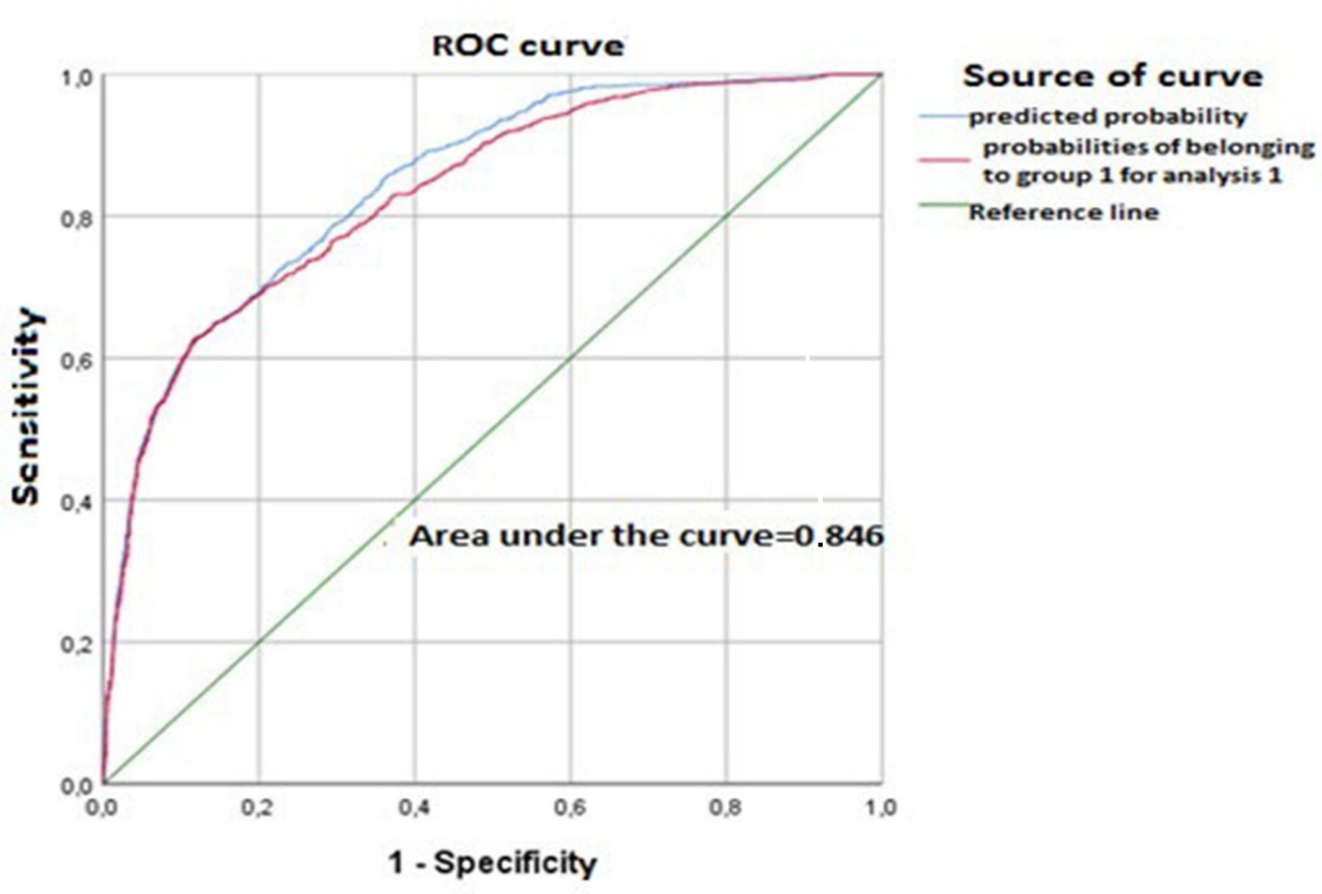
ROC curve plot showing the symptom-based prediction of SARS-CoV-2 positivity using the binary logistic regression model. The calculated area under the curve (performance index) is 0.846. Sensitivity determines the true positive rate. Specificity determines the false negative rate. The ROC curve thus plots the true-positive rate against the false-positive rate.

Symptom-based prediction of positivity by RT-PCR revealed a performance index of 0.846, which was derived from the area under the curve using the binary logistic regression model [95 % CI = (0.832; 0.859); *P*<0.001] (Fig. 4).

### Risk factors associated with SARS-CoV-2 infection

Table 2 shows the independent risk factors for SARS-CoV-2 infection according to the results of univariate and multivariate logistic regression analyses with adjusted ORs. Patients in the age groups 11–20 years, 21–30 years and 31–40 years were more likely to be infected with SARS-CoV-2 than patients in the age group ≤10 years and 91–100 years (Table 2). In addition, Individuals who were screened for suspected COVID-19 infection (adjusted OR=2.156; *P<*0.001) and contact cases (adjusted OR=1.447; *P*=0.063) were also more likely to be COVID-19-positive. Further multivariate logistic regression analysis of the most severe clinical symptoms reported by the patients revealed that patients suffering from loss of taste and/or smell (adjusted OR=10.484; *P<*0.001) had a 10-fold higher risk of being COVID-19-positive. Fever, fatigue and cough were also functional signs of probable infection (Table 2).

**Table 2.** Risk factors (OR and adjusted OR) associated with COVID-19

	Univariate analyses		Multivariate analysis	
Variables	OR (95 % CI)	*P*-value	Adjusted OR (95 % CI)	*P*-value
**Sex of patients**				
Male vs female	0.761 (0.659; 0.878)	*P<*0.001	0.835 (0.703;0.9901)	0.038
**Age group by categories: ref: 91 to 100** **years**				
≤10	2.025 (0.227;18.074)	0.527	0.788 (0.362;1.716)	0.548
11 to 20	1.521 (0.188;12.289)	0.693	1.813 [1.239; 2.654]	0.002
21 to 30	1.664 (0.209;13.241)	0.630	1.798 (1.419; 2.277)	<0.001
31 to 40	1.664 (0.260;16.452)	0.491	1.322 (1.065;1.641)	0.011
41 to 50	2.260 (0.284;17.975)	0.440	1.041 (0.834;1.298)	0.724
51 to 60	3.016 (0.379; 23.967)	0.296	0.652 (0.527; 0.805)	<0.001
61 to 70	3.883 (0.487; 30.929)	0.200	0.547 (0.430;0.695)	<0.001
71 to 80	3.815 (0.475; 30.628)	0.207	0.648 (0.469; 0.897)	0.009
81 to 90	4.078 (0.493; 33.707)	0.192	0.715 (0.419;1.223)	0.221
91 to 100	–	<0.001	–	<0.001
**Symptoms**				
**Fever**: yes vs no	11.471 (9.347;14.077)	<0.001	4.524 (3.496; 5.853)	<0.001
**Fatigue**: yes vs no	7.071 (5.985; 8.355)	<0.001	2.383 (1.891; 3.003)	<0.001
**Cough**: yes vs no	6.519 (5.367; 7.919)	<0.001	1.860 (1.431; 2.419)	<0.001
**Diarrhoea**: yes vs no	5.153 (3.795; 6.996)	<0.001	0.445 (0.285; 0.695)	<0.001
**Loss of taste and/or smell**: yes vs no	18.125 (13.792; 23.817)	<0.001	10.484 (7.740; 14.202)	<0.001
**Other symptoms**: yes vs no	3.329 [2.619; 4.232)	<0.001	1.159 (0.841; 1.596)	0.367
**Reasons for testing**				
**Contact cases**: yes vs no	1.451 (1.248; 1.687)	<0.001	1.447 (0.981; 2.135)	0.063
**Screening**: yes vs no	3.258 (2.814; 3.772)	<0.001	2.156 (1.470; 3.160)	<0.001
**Travel**: yes vs no	0.034 (0.022; 0.054)	<0.001	0.061 (0.034; 0.108)	<0.001
**Other reasons for screening**: yes vs no	0.889 (0.610; 1.294)	0.539	1.412 (0.900;2.216)	0.133

### Importance of the cycle threshold (*C*_t_) in the diagnosis of COVID-19 by RT-PCR

The Argene amplification kit revealed that 55.7 % of the identified positive cases were positive for both the *RdRp* and *N* genes (RT-PCR). The median and interquartile ranges (IQRs) for the *RdRp* gene and the *N* gene were 25 (21–28) and 22 (18–26), respectively. In addition 3.3 % of the positive cases were only positive for the *N* gene, and 0.5 % were only positive for the *E* gene (RT-PCR2) (median and IQR: 35 (34.5–36.5).

**Fig. 5.**
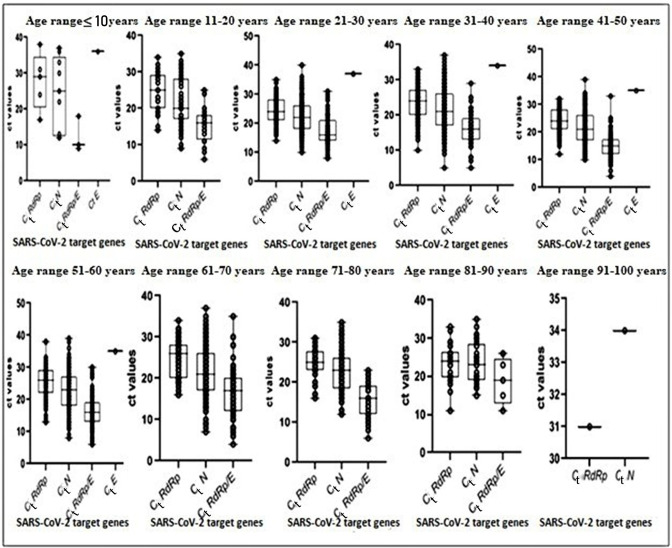
Distribution of *C*_t_ values of different target genes according to age group. The box plots show the dispersion and frequency density of *C*_t_ values (black circle) of the virus target genes in positive patients. The centre lines of the box plots represent the median *C*_t_ values. The limits of box plots indicate the first (25 %) and third quartiles (75 %). *C*_t_ values are indicators of viral load; Eyre *et al*. 2022 describe details of equivalent viral loads in copies ml^−1^ = (log_10_ viral load=12.0–0.328*×C*_t_) [23].

Using the GeneProof kit, 40.5 % of positive cases were positive for the two main target genes *RdRp/E* and *N.* The median *C*_t_ values of the *RdRp/E* gene and *N* gene were 16 (IQR: 13–19) and 22 (IQR: 18–26), respectively; 0.5 % of cases with a RT-PCR-positive status were found to be positive by targeting the *RdRp/E* gene. It is important to note that we used the inverse of *C*_t_ values as an indicator of viral load. According to Eyre *et al*. 2022, the equivalent viral load in copies ml^−1^ is described according to the following formula: (log_10_ viral load=12.0–0.328×*C*_t_) [23]. Fig. 5 shows the distribution of *C*_t_ values of different genes according to age group. The median *C*_t_ values and IQRs of the targeted genes according to the age group of the COVID-19-positive patients were as follows: 21–30 years [*C*_t_ *RdRp*: 24 (21–28), *C*_t_ *N*: 22 (18–26), *C*_t_ *RdRp/E*: 16 (14–21), *C*_t_ *E*: (37)]; 31–40 years [*C*_t_ *RdRp*: 24 (20–27), *C*_t_ *N*: 21 (17–26), *C*_t_ *RdRp/E*: 16 (13–19), *C*_t_ *E*: (34)]; 41–50 years [*C*_t_ *RdRp*: 24 (21–28), *C*_t_ *N*: 21 (17.25–26), *C*_t_ *RdRp/E*: 15.5 (12–18), *C*_t_ *E*: (35)], 51–60 years [*C*_t_ *RdRp*: 26 (22–29), *C*_t_ *N*: 23 (18–27), *C*_t_ *RdRp/E*: 16 (13–19), *C*_t_ *E*: (35)], 61–70 years [*C*_t_ *RdRp*: 26 (20–28),*C*_t_ *N*: 21 (17–26), *C*_t_ *RdRp/E*: 17 (12–20)] and 71–80 years [*C*_t_ *RdRp*: 25 (23–27.75), *C*_t_ *N*: 23 (26–28), *C*_t_ *RdRp/E*: 16 (12–19)]. These *C*_t_ values reflect moderately high and moderately low viral loads (high: 10≤*C*_t_<20; low: *C*_t_ values (20–30)) (*P*<0.001). In other words, there was significant variation in the *C*_t_ values of the different target genes according to age group.

A correlation analysis showed the absence of a linear relationship between the *C*_t_ values of the different genes and the fraction of positive cases in the study population: *C*_t_ *RdRp* (r=−0.19; *P<*0.001), *C*_t_ *N* (r=−0.37; *P<*0.001), *C*_t_ *RdRp/E* (r=−0.21; *P<*0.0001), except for *C*_t_ *E* (r=0.38; *P=*0.096). Correlation analysis showed no linear correlation between age and *C*_t_ value: *C*_t_ *RdRp* (r=0.031; *P=*0.473), *C*_t_ *N* (r=0.026; *P=*0.416), *C*_t_ *RdRp/E* (r=−0.003; *P=*0.956) and *C*_t_ *E* (r=0.255; *P=*0.277), with less variation in the *C*_t_ values of the targeted genes as a function of age; however, significant variation was observed between the *C*_t_ values of different genes and age, i.e. there was a significant difference between the *C*_t_ values of *RdRp/E* and *N* and between those of *RdRp/E* and *RdRp* and *E*.

## Discussion

The current SARS-CoV-2 outbreak is the third epidemic attributed to coronavirus in the 21st century, characterized by high contagiousness compared to SARS-CoV-1 and MERS-CoV, which appeared in 2002 and 2012, respectively [24]. The variety of clinical presentations of the disease and the absence of specificity of the signs presented by the patients impose a mandatory recourse to PCR for confirmation of diagnosis [25].

In our study, women were more prone to infection than men, which is consistent with the results of previous studies, such as the one conducted by the Korean Society of Infectious Diseases and the one reported by Rozenberg *et al*. [26, 27]. Nevertheless, the higher incidence of infection among women in our study may be related to the fact that the majority of patients sampled were women.

Age is a variable motor of epidemic trajectories of COVID-19 worldwide, since the rate of infection is highly dependent on the age pyramid [28]. In other words, countries with a large older population may experience large and rapid epidemics in the absence of response and intervention. In addition, the mean age of COVID-19-positive patients in our study was 47.5±18 years. The age group 51–60 years was the most infected in this study, with a proportion of 19.1%, in agreement with the observations of Tian *et al*. [29] as well as hat was reported in Wuhan, PR China [29–31].The mean age of infection in our study shows that mostly young people were affected, as highlighted elsewhere [32]. Older individuals (>60 years) were less likely to be infected with COVID-19 than younger individuals (Table 2). As Morocco has a younger population, it is normal that the number of cases is higher among those under 60 years old. Other reasons for this observation could be the fact that some youths may be asymptomatic and are actively working, with more interactions with other people and outdoor engagements and less observance of safety protocol measures, which would be the cause of the increase in cases. This is why we believed that younger people could be the main reservoir for the spread of the virus within the community. This observation is corroborated by data from Brazil [33]. Nevertheless, all age groups are at risk of COVID-19, and in terms of disease severity, the elderly are at greater risk because of the physiological changes that accompany aging, a weakened immune system and potential underlying health problems [17].

If the primary manifestations of the disease were dominated by respiratory signs, current research attributes other clinical manifestations to it. In our study, the quartet of loss of taste/smell–fever–fatigue–cough dominated the clinical manifestations observed. These symptoms were similar to those reported by the CDC and to those reported in previous studies [15, 17, 34]. The multivariate logistic regression adjustment model showed that patients who developed loss of taste and/or smell had more than a 10-fold multivariate probability of testing positive for COVID-19 [adjusted OR=10.484 with 95 % CI (7.740; 14.202); *P<*0.001]. Our results showing that loss of taste and/or smell is a potential predictor of COVID-19 corroborate numerous studies in the UK and USA [35]. In addition to other more established symptoms, those who developed a fever had a fourfold higher probability of testing positive (Table 2). Fever appears to be common during the course of COVID-19 and has therefore become a widely used screening tool in crowded public places. The study population that developed fatigue had a twofold higher probability of having a positive result. Although it is one of the factors suggestive of COVID-19 infection, it is considered by some authors to be a non-specific symptom. Patients with a cough were also included among those at risk (adjusted OR=1.860).These results corroborate those of many other studies [28, 35–39].Our results are similar to those of Weiss *et al*. [40], who reported that although fatigue, fever and cough are significantly associated with an increased risk of SARS-CoV-2 infection, these symptoms are a common feature of other types of respiratory infections and are thus not specific to COVID-19 and thus are less important than loss of taste and/or smell for COVID-19 surveillance [40]. Pairwise correlation analysis between the reported symptoms revealed highly significant *P*-values (*P<*0.001) (Fig. 3). The association between symptoms could be used to identify specific COVID-19 statuses; hence, documentation of these symptoms could make the diagnosis of COVID-19 more accurate than the diagnosis of other viral infections.

Interestingly, according to Chinese data, children account for 1 % of SARS-CoV-2 infections regardless of age. This observation interferes with our data (Table 1) [41]. Among 4569 samples analysed in our study, 34 were children, with 79.4 % (*n*=27) asymptomatic cases and 20.6 % (*n*=7) symptomatic cases identified. However, the five positive cases in children were all asymptomatic. This observation corroborates with the study of Chekhlabi *et al*. and the hypothesis of He *et al*. 2020 suggesting that the main reason why children with COVID-19 are not detected is because they remain asymptomatic with a benign course of the disease [42, 43]. On the other hand, it may be due in part to host (children) factors, such as angiotensin-converting enzyme 2 (ACE2) and transmembrane serine protease 2 (TMPRSS 2), which have been reported to be involved in the pathogenesis and severity of the disease. The activities of ACE2 and TMPRSS 2 are significantly weaker in children than in adults, which is consistent with the fact that the immune systems of children are less mature than those of adults, resulting in a less abrupt immune response in children during SARS-CoV-2 infection [42–44].

The *C*_t_ values (viral load) of the subjects whose samples were analysed were also taken into consideration. They are important markers in guiding antiviral treatment and could explain the pattern of recovery and transmission of infections [17]. Some studies have suggested that children have higher viral loads than adults [17]. However, in our study, viral load did not appear to correlate with age; correlation analysis between age and *C*_t_ values revealed no significant linear correlation, which explains why older patients and children had approximately similar viral loads. Our observations are similar to those of Refs [45]. However, some authors have reported that a high SARS-CoV-2 viral load is associated with a poor clinical outcome [46]. In addition, some documented studies indicate that early *C*_t_ values obtained from a nasopharyngeal swab will correlate with disease susceptibility, age and comorbidities, will be associated with potential differential expression of the ACE2 receptor and various clinical signs, and will predict the degree of infectivity, severity and survival in symptomatic patients [47]. An assessment of viral loads based on *C*_t_ values between symptomatic and asymptomatic subjects was also analysed in our study. Patients with high viral loads (10≤*C*_t_<20) were mostly symptomatic, with a statistically significant difference compared to asymptomatic subjects (*P*<0.001). The majority of age groups in this study had moderate viral loads. Our results agree with those of several documented studies [17, 48]. In addition, the relevance and clinical utility of estimating viral load measurements in this study allowed us to specifically classify patients into three categories: those in the early stage of disease with high viral load (10≤*C*_t_<20), those in the moderate stage of disease with moderate viral load (*C*_t_ 20–30),and those in third stage disease when the viral load tends to decrease (low viral load) (*C*_t_>30). However, some studies have reported that the interpretation of results in patients with higher *C*_t_ values in the presymptomatic, asymptomatic, or early post-symptomatic phases should be interpreted with caution, as high *C*_t_ values do not necessarily imply an old infection if the onset of symptoms is unknown [21]. A predominance of *C*_t_ values between 20 and 30, with median values and IQR fluctuating between 22 (18–26) and 25 (21–28) with the Argene kit and 16 (13–19) and 22 (18–26) with the GeneProof kit, in our study reflected a high prevalence of SARS-CoV-2 contamination at the community level (Fig. 5). *C*_t_ values are therefore relevant viral biomarkers in viral diagnosis and would allow us to orientate the adapted treatment according to the medical history of each patient.

According to our analysis, coronavirus disease (COVID-19) infection showed unprecedented heterogeneity with respect to who was infected and in terms of symptoms. All age groups, irrespective of sex, were affected, and symptoms varied widely from one person to another. Nevertheless, some positive patients were asymptomatic (*n*=327; 33.8 %) and others symptomatic (*n*=640; 66.2 %) with a *P*-value <0.001. Thus, in contrast to other studies [15], the majority of patients in our study were symptomatic. It is important to note that asymptomatic patients play a significant role in viral transmission in the community; therefore, it is essential to know the proportion of asymptomatic infections in the community to better predict the potential for virus transmission and curb the spread of infection [17]. Our study showed that there were 967 positive SARS-CoV-2 cases among the 4965 samples analysed, representing a prevalence of 21.2 %. We conclude that the observed prevalence was higher than the current national prevalence of 8.8 % (as of 9 May 2021) as well as the prevalence reported in many other studies [17, 49, 50]. Nevertheless, suspected COVID-19 (adjusted OR=2.156; *P*<0.001) and contact cases (adjusted OR=1.447; *P*=0.063) were likely to be positive for SARS-CoV-2. This study has some notable limitations. There was a lack of reporting of the date for the onset of symptoms versus the date when clinical samples were obtained (interval) and for the interval between contact with a confirmed case and testing, as well as the first sign/symptom and follow-up/evolution of confirmed cases. In other words, this study was not able to follow up the presumed presymptomatic or asymptomatic people to ascertain if they developed symptoms along with the course of the ailment. On the other hand, an analysis of the dynamics of SARS-CoV-2 target genes from the onset of infection to eradication of the virus to explore the utility of genes persisting (duration) after infection could not be analysed. Viral culture was not performed to determine at what *C*_t_ value a patient was no longer at risk of infecting others. The samples included in this study were tested by different commercial kits where some gene targets generally gave lower *C*_t_ values than others, which would cause variation in the *C*_t_ value. On the other hand, different RNA extraction methods with different extraction volumes could produce lower *C*_t_ values.

### Conclusion

In this study, there was a higher prevalence of COVID-19 than the national prevalence. However, young adults (<60 years) were more likely to contract COVID-19 than other age groups. Particular attention was paid to functional clinical signs, including loss of taste and/or smell, which, among other functional signs (fever, fatigue, cough), was the strongest predictor of SARS-CoV-2 infection in patients with suspected COVID-19. It would be wise to take these symptoms into account at diagnosis to minimize the delay in the diagnosis. On the other hand, the interpretation of results following SARS-CoV-2 infection associated with the clinical context and *C*_t_ values was defined as a relevant algorithm for COVID-19 diagnosis in this study. This association between *C*_t_ values (viral load) and clinical context would allow the establishment of an adapted management scheme for patients according to their medical history. In addition, this study provided significant results that will be used to complement previous studies conducted in Morocco to monitor the epidemiological situation while improving the quality of diagnosis (early, accurate and rapid diagnosis, followed by early isolation) and the identification of epidemio-clinical characteristics for a better understanding of clinical outcomes to overcome the pandemic.
